# Agomelatine's antiglycoxidative action—*In vitro* and *in silico* research and systematic literature review

**DOI:** 10.3389/fpsyt.2023.1164459

**Published:** 2023-04-25

**Authors:** Miłosz Nesterowicz, Kamil Klaudiusz Lauko, Małgorzata Żendzian-Piotrowska, Jerzy Robert Ładny, Anna Zalewska, Mateusz Maciejczyk

**Affiliations:** ^1^Students' Scientific Club “Biochemistry of Civilization Diseases” at the Department of Hygiene, Epidemiology and Ergonomics, Medical University of Bialystok, Białystok, Poland; ^2^Department of Hygiene, Epidemiology and Ergonomics, Medical University of Bialystok, Białystok, Poland; ^3^1st Department of General Surgery and Endocrinology, Medical University of Bialystok, Białystok, Poland; ^4^Independent Laboratory of Experimental Dentistry, Medical University of Bialystok, Białystok, Poland

**Keywords:** agomelatine, antiglycation activity, antioxidants, carbonyl stress, depression, antiglycoxidation action, oxidative stress, reactive oxygen species

## Abstract

**Introduction:**

Agomelatine is an atypical antidepressant drug enhancing norepinephrine and dopamine liberation; nevertheless, additional mechanisms are considered for the drug's pharmacological action. Since protein glycoxidation plays a crucial role in depression pathogenesis, agomelatine's impact on carbonyl/oxidative stress was the research purpose.

**Methods:**

Reactive oxygen species scavenging (hydroxyl radical, hydrogen peroxide, and nitrogen oxide) and antioxidant capacity (2,2-diphenyl-1-picrylhydrazyl radical and ferrous ion chelating assays) of agomelatine were marked. Agomelatine's antiglycoxidation properties were assayed in sugars (glucose, fructose, and galactose) and aldehydes- (glyoxal and methylglyoxal) glycated bovine serum albumin (BSA). Aminoguanidine and α-lipoic acid were used as standard glycation/oxidation inhibitors.

**Results:**

Agomelatine did not show meaningful scavenging/antioxidant capacity vs. standards. Sugars/aldehydes increased glycation (↑kynurenine, ↑N-formylkynurenine, ↑dityrosine, ↑advanced glycation end products, and ↑β-amyloid) and oxidation (↑protein carbonyls and ↑advanced oxidation protein products) parameters in addition to BSA. Standards restored BSA baselines of glycation and oxidation markers, unlike agomelatine which sometimes even intensifies glycation above BSA + glycators levels. Molecular docking analysis of agomelatine in BSA demonstrated its very weak binding affinity.

**Discussion:**

Agomelatine's very low affinity to the BSA could proclaim non-specific bonding and simplify attachment of glycation factors. Thereby, the drug may stimulate brain adaptation to carbonyl/oxidative stress as the systematic review indicates. Moreover, the drug's active metabolites could exert an antiglycoxidative effect.

## 1. Introduction

Depression is the leading cause of disability/incapacity and the most common mental disorder. Currently, 350 million people worldwide suffer from depression (1). Such a high number of cases is due to modern lifestyles—e.g., its fast pace, the overload of responsibilities, and an imbalance between work and leisure. Depression is, therefore, a disease of civilization (2). It is diagnosed most often in people aged between 20 and 40 (1). Depression is a significant risk factor for suicide and, therefore, contributes to 1 million deaths per year worldwide. It is estimated that at least half of the disease cases remain undiagnosed, and only half of the patients diagnosed with depression receive adequate treatment (1, 3). However, pharmacotherapy for depression is characterized by increasing effectiveness and limiting side effects. The most common division of antidepressants, precisely by mechanism, includes selective serotonin reuptake inhibitors (SSRIs), serotonin and norepinephrine reuptake inhibitors (SNRIs), tricyclic antidepressants (TCAs), and monoamine oxidase inhibitors (MAOIs) (4). SSRIs (e.g., sertraline, paroxetine, fluvoxamine, fluoxetine, escitalopram, and citalopram) block the transport of serotonin across the presynaptic membrane back into the nerve cell, increasing its concentration in the brain. SNRIs are another group that includes venlafaxine, milnacipran, and duloxetine. They increase brain levels of serotonin and norepinephrine and exhibit analgesic effects. TCAs, like SNRIs, inhibit the reuptake of norepinephrine and serotonin and have analgesic action. These include amitriptyline, desipramine, imipramine, clomipramine, nortriptyline, and doxepin. The only MAOI currently used to treat depression is moclobemide. It reversibly blocks type A monoamine oxidase (MAO-A), the enzyme responsible for breaking down serotonin, norepinephrine, and dopamine. However, these drugs are not without many side effects, such as pain and dizziness, migraine, nausea, gastrointestinal problems, increased sweating, back pain, fatigue, increased liver enzymes, and anxiety (4, 5).

**Figure 1:**
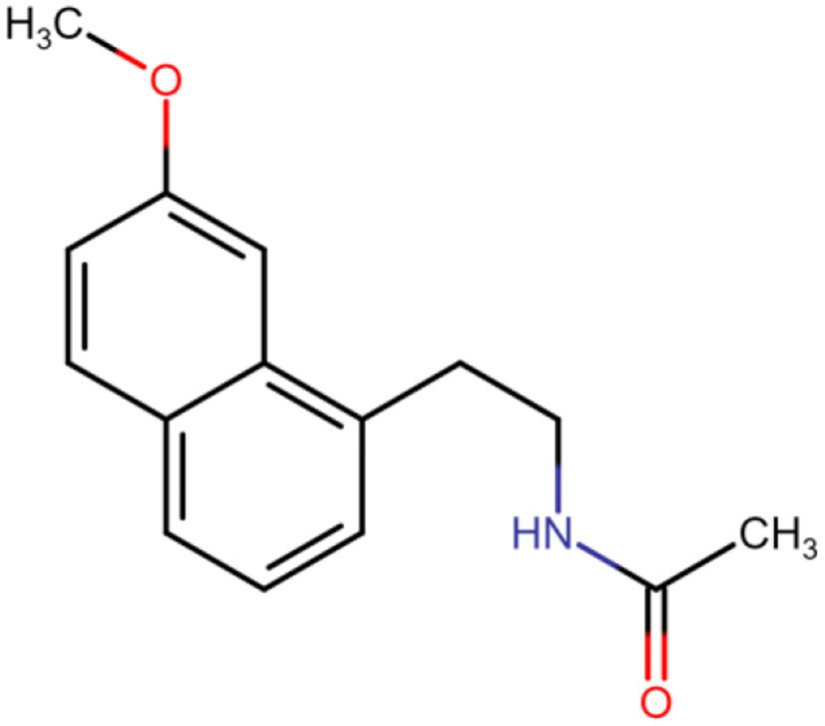
Chemical structure of agomelatine.

A drug that stands out in this regard is agomelatine (N-[2-(7-methoxynaphthalen-1-yl)ethyl]acetamide; C_15_H_17_NO_2_; Figure 1) (6). Agomelatine increases the release of norepinephrine and dopamine and stimulates MT_1_ and MT_2_ melatonergic receptors. In this way, agomelatine helps regulate sleep–wake rhythms, which are often disrupted in depressed patients (insomnia and excessive sleepiness). Agomelatine is classified as an atypical antidepressant, and its mechanism of action has not been fully understood (6, 7). The drug is absorbed rapidly and well (≥80%). Bioavailability amounts to <5%. Consumption of a standard or high-fat meal does not alter bioavailability or absorption rate. Bioavailability increases when taking oral contraceptives and decreases in smokers. Agomelatine is 95% bound to plasma proteins. The drug is metabolized in the liver and excreted mainly by the kidneys (80%). Severe renal insufficiency does not significantly affect pharmacokinetic parameters, while impaired liver function increases exposure to the drug (6–9). Agomelatine is used to treat not only depression but also sleep disturbances (10).

The brain is an organ eminently susceptible to oxidative damage. Cell membranes of neurons contain significant amounts of polyunsaturated fatty acids, making them easily oxidized by reactive oxygen species (ROS). The accumulation of transition metals and low levels of antioxidant enzyme activities accelerate oxidation as well (11). The brain is also susceptible to glycemic fluctuations. High glucose concentrations in neurons induce oxidative stress and increase the formation of advanced glycation end products (AGEs). AGEs induce NADPH oxidase (NOX) to produce ROS by activation of the receptor for advanced glycation end products (RAGE), as well as to stimulate the formation of cytotoxic β-amyloid (βA) and ceramide, inducing neuronal death. βA, through AGEs signaling, can contribute to the phosphorylation of mitogen-activated protein kinase (MAPK) and, thus, to the atrophy of synapses (11–13). Therefore, it should be no surprise that 11% of people with diabetes meet the criteria for a diagnosis of depression, and 31% report a history of depressive symptoms (14). Carbonyl stress also plays a vital role in sleep–wake disorders. Systemic inflammation activates microglia cells and astrocytes in brain regions that regulate circadian rhythms (15). These cells can secrete proinflammatory cytokines [such as tumor necrosis factor-α (TNF-α) or interleukin-1β (IL-1β)], nitric oxide (NO•), and gliotransmitters (16). These can affect the expression of key genes regulating the circadian rhythms, such as circadian locomotor output cycles kaput (CLOCK) (10, 15). Moreover, sleep disturbances can aggravate oxidative/nitrosative injury, peripheral immune activation, and neuroinflammation in a vicious cycle mechanism (10, 15, 16). Thus, it is unsurprising that new antidepressants are still being sought to inhibit oxidative stress and protein glycation.

The literature seems to confirm the antioxidant effect of agomelatine. Among other things, the drug scavenged ROS, reduced levels of oxidation-promoting enzymes, increased concentrations of enzymatic and non-enzymatic antioxidants, or modulated the expression of genes related to redox homeostasis. However, studies on the counteracting of protein glycation by agomelatine are scarce (17–20). Carbonyl stress is an essential pathogenetic component of depression (15), and agomelatine may also act through an unknown mechanism to inhibit oxidative stress and protein glycation. Therefore, our study was the first to evaluate the antiglycoxidative effect of agomelatine comprehensively.

## 2. Materials and methods

### 2.1. Systematic review

The literature review was conducted between 1993 and October 2022 following the PRISMA guide with PubMed (National Library of Medicine, Washington, DC, USA), Scopus (Elsevier B.V.), and Web of Science Core Collection (WOS) databases. The search strategy was conducted with the usage of Medical Subject Headings (MeSH) terms. The obtainable references were trawled through using keywords: (agomelatine and oxidative stress), (agomelatine and ROS), (agomelatine and antioxidant), (agomelatine and protein glycation) as well as (agomelatine and antiglycation properties). Inclusion and exclusion criteria are presented in Table 1.

**Table 1:** Analyzed publications' inclusion and exclusion criteria.

**Inclusion criteria**	**Exclusion criteria**
Publications solely in English	Publications in other languages
Antiglycoxidative activity of agomelatine articles	Articles not describing the antiglycoxidative activity of agomelatine
Manuscripts pertinent to human *in vivo* and *in vitro* experiments	Review manuscripts, surveys, as well as case descriptions

Two scientists (M.N. and K.K.L.) investigated the initial data by independently analyzing the titles of articles and abstracts. Afterward, all previously selected manuscripts were evaluated by two other authors (M.M. and M.Z.P.). Next, the research articles which met the set criteria were chosen for the final analysis. The researchers' reliability level was assessed with Cohen's kappa coefficient (κ), which equaled κ = 0.97. All articles were evaluated methodologically, and the following variables were assayed: authors, publication year, study design, experiment population size, inclusion and exclusion criteria, experiment length, and endpoints.

### 2.2. Reagents and equipment

All analytical grade reagents were purchased from Sigma-Aldrich (Numbrecht, Germany/Saint Louis, MO, USA). First, all solutions were sterilized by filtration through 0.2 mm membrane filters directly before use.

To differentiate the results obtained for agomelatine, aminoguanidine was used, as a known protein oxidation inhibitor, and α-lipoic acid (ALA), as an antioxidant. The concentration of all additives was 1 mM (based on *in vitro*, kinetic studies), in proportion to the high concentrations of the glycating agents (21–26).

The absorbance and fluorescence were assessed with an M200 PRO multimode microplate reader (Tecan Group Ltd., Männedorf, Switzerland).

### 2.3. Scavenging of reactive oxygen species

Agomelatine's ability to neutralize ROS was conducted in duplicate samples in three independent experiments.

#### 2.3.1. Hydroxyl radical scavenging

The scavenging activity of HO• was measured via the modified assay described by Su et al. (27). First, 0.25 mL of FeSO_4_ (8 mM), 0.4 mL of H_2_O_2_ (6 mM), 0.25 mL of distilled water, 0.5 mL of the samples (terminal strength of 1 mM), and 0.2 mL of sodium salicylate (20 mM) were all mixed and next incubated at 37°C for 1 h. The absorbance of the reaction was assessed spectrophotometrically at 562 nm wavelength. The scavenging of HO• (%) was assessed by the formula: [1 – (A_1_ – A_2_)/A_0_] × 100%, where A_0_–absorbance of the control (without additives), A_1_–after the drugs were mixed with the drugs, and A_2_–with no sodium salicylate (27).

#### 2.3.2. Hydrogen peroxide scavenging

The evaluation of H_2_O_2_ scavenging activity was conducted in compliance with the procedure by Kwon et al. (28). Initially, to obtain a solution of ferrous ion oxidation-xylenol orange (FOX), butylated hydroxytoluene (BHT) (87.3 mg), H_2_SO_4_ (10 μL), xylenol orange (7.6 mg), and ferrous ammonium sulfate (10 mg) were mixed in 100 mL of 90% methanol–water solution. After that, H_2_O_2_ (50 mM) and the samples (terminal strength of 1 mM) were mixed (1:1, v/v) and incubated for 30 min at room temperature. Next, 10 μL of high-performance liquid chromatography (HPLC)-grade methanol was carefully mixed with 90 μL of the abovementioned solution. Next, the FOX reagent (0.9 mL) was introduced to the above mixture, where it was vortexed and incubated for 30 min at room temperature. Spectrophotometrically, at a wavelength of 560 nm, the absorbance of the reaction product, ferric-xylenol orange complex, was assessed. The scavenging of H_2_O_2_ (%) was evaluated by the formula: [1 – f(A_1_ – A_2_)/A_0_g] × 100% (A_0−−_control absorbance (without additives), A_1_–post-addition of the drugs, and A_2_–without the FOX reagent) (28).

#### 2.3.3. Nitric oxide scavenging

Phosphate-buffered saline (100 μL) was mixed including sodium nitroprusside (5 mM) to 50 μL of samples. Next, the mixture was incubated for 150 min at 25°C. Then, Griess reagent (150 μL) was added to the reaction mixture [Griess reagent including sulfanilamide (1%), H_3_PO_4_ (2%), and N-(1-naphthyl)ethylenediamine] (0.1%)]. Chromophore was released as a result of nitrite diazotization by sulfanilamide with its conjugation with N-(1-naphthyl) ethylenediamine. The assessment of product absorbance was conducted using a spectrophotometer at a wavelength of 546 nm. The scavenging capacity was determined according to the formula: [1 – (*A*_1_/*A*_2_)] × 100% [A_1_–sample absorbance after the drugs are mixed, *A*_2_—absorbance (without the Griess reagent)] (29).

### 2.4. Antioxidant capacity

The antioxidant capacity of agomelatine was tested in duplicate samples in three independent experiments.

#### 2.4.1. Scavenging capacity of 2,2-diphenyl-1-picrylhydrazyl radical

The assessment of free radical scavenging activity was performed based on the decolorization of DPPH radical (DPPH•) according to Kwon et al. (28). A diluted sample (30 μL) was mixed with DPPH solution (180 μL) (0.13 mg/mL). After that, methanol was added to the volume of 210 μL. The DPPH solution was utilized as a control. Next, the reaction mixture was incubated for 20 min, and the absorbance was measured at 518 nm wavelength. DPPH• elimination was calculated using the following formula: [(A_blank_ – A_sample_)/A_blank_] 100%, where A_blank_–absorbance of the blank DPPH solution, and A_sample_–DPPH absorbance of the sample mixture (28).

#### 2.4.2. Ferrous ion chelating

By calculating the decrease in the generation of the Fe^2+^-ferrozine complex, FIC was measured. Approximately 18 μL of FeCl_2_ (0.6 mM) and 16 μL of CH_3_OH were mixed with 90 μL of samples (terminal strength of 1 mM) or BHT (control). Next, the mixture was incubated at room temperature for 10 min. Quickly, 18 μL of solution of ferrozine (5 mM) was added. The mixture was incubated at room temperature for an extra 5 min. A spectrophotometer was used to calculate the absorbance at a wavelength of 562 nm. The percentage decrease in absorbance of the control allowed us to express FIC (28).

### 2.5. Experimental model

The glycation of bovine serum albumin (BSA) was performed according to a previously published method (21, 22, 24–26). Promptly, BSA (>98% purity; protease- and fatty acid-free; 90 μmol/L) was dissolved in sodium phosphate buffer (0.1 M, pH 7.4), which included 0.02% sodium azide as a preservative. As glycating agents, sugars [glucose (Glu), fructose (Fru), and galactose (Gal); 10 mM) and aldehydes (glyoxal (GO) and methylglyoxal (MGO); 2.5 mM] were used. Incubation was performed in closed vials in the dark, with ongoing shaking (50 rpm, 37°C; 6 days with sugars and 12 h with aldehydes). Despite the fact that the concentrations of glycators were much higher than their physiological levels, they are useful for modeling in a comparatively short time the physiological processes occurring in the body over several months. Such conditions are applied routinely to determine the antiglycooxidant properties of new substances (21, 22, 24–26, 30). The concentration of all additives was 1 mM (based on *in vitro* kinetic studies), in proportion to the high level of the glycating agents (21, 22, 24–26, 30). The study was performed in three independent experiments, each time in duplicate.

### 2.6. Carbonyl stress products

#### 2.6.1. Amino acid glycation products

Kynurenine (KN), N-formylkynurenine (NFK), and dityrosine (DT) were evaluated spectrofluorimetrically at the following fluorescence emission and excitation wavelengths: 365/480, 325/434, and 330/415 nm, respectively. Before the study, the samples were diluted with 0.1 M H_2_SO_4_ (1:5, v/v). Next, according to the fluorescence of 0.1 mg/mL quinine sulfate solution in 0.1 M H_2_SO_4_, the results were standardized (31).

#### 2.6.2. β-Amyloid

Thioflavin T was examined to mark fluorescence emitted during the binding of amyloid fibrils/oligomers to thioflavin T. Thioflavin T (10 μL) and samples (90 μL) were mixed and placed on a microplate. Fluorescence intensity was quantified at a 385/485 nm wavelength (32).

#### 2.6.3. Advanced glycation end products

A spectrofluorometer was used to examine the content of AGEs. AGE-specific fluorescence was analyzed at 440/370 nm wavelength. Before the study, the assayed samples were diluted with PBS (1:5, v/v).

### 2.7. Oxidative stress products

#### 2.7.1. Protein carbonyls

To determine the concentration of PCs, a reaction with 2,4-dinitrophenylhydrazine (2,4-DNPH) and carbonyls in oxidation-damaged proteins was performed. The spectrophotometer, at a 355 nm wavelength, allowed us to determine the reaction product absorbance. The absorption coefficient for 2,4-DNPH (22 000 M^−1^cm^−1^) was used (33).

#### 2.7.2. Advanced oxidation protein products

A spectrophotometric assay was performed to evaluate the level of AOPPs. Studied samples (200 μL) were diluted with PBS at a ratio of 1:5 (v/v). Next, the prepared samples and standard solutions (0–100 μmol/L) as well as a blank PBS solution (200 μL) were allocated to a 96-well microplate. Subsequently, 10 μL of KI (strength of 1.16 M) and 20 μL of CH_3_COOH were inserted into the wells. The microplate reader, at a 340 nm wavelength, allowed us to rapidly assay the absorbance, in contrast with the blank solution (200 μL PBS, 10 μL KI, and 20 μL CH_3_COOH). A linear absorbance was manifested in the range of 0–100 μmol/L by the chloramine T solutions (34).

### 2.8. Molecular docking analysis

Molecular docking is a study that is used in the *in silico* technique of foreseeing the preferred position of a ligand after binding to a macromolecule (commonly a protein). In our study, BSA was used as a receptor in an interaction study with the agomelatine molecule. A 3D structure of BSA (PDB ID: 4F5S) was transferred from the Protein Data Bank (PDB) (https://www.rcsb.org/) in the.pdb format. The crystal structure was established with the X-ray diffraction method at a resolution value of 2.47 Å. The 3D structure of agomelatine (PubChem CID: 82148) was downloaded from the National Library of Medicine website (https://pubchem.ncbi.nlm.nih.gov/compound/Agomelatine) as an.sdf file. All the water molecules and the addition of polar hydrogens as well as Kollman charges were deleted using the AutoDock MGL Tools. The processed protein particle was prepared and saved in.pdbqt format. We set the exhaustiveness parameter value at the level of 8. Molecular docking simulation was exercised by AutoDock Vina (grid size: 40 × 40 × 40; spacing located at 34.885, 23.976, and 98.792). To visualize the molecular docking, PyMOL 2.5 program was used (26).

### 2.9. Statistical analysis

The statistical analysis was conducted using GraphPad Prism 8.3.0 (GraphPad Software, La Jolla, CA, USA). The results were expressed as a percentage of the respective control values [BSA + glycation agent (Glc, Fru, Gal, GO, and MGO)]. The evaluation of the result distribution was conducted using the D'Agostino-Pearson and Shapiro–Wilk tests. The homogeneity of variance was checked by Levine's test. The differences between groups were evaluated by one-way analysis of variance ANOVA followed by Tukey's *post-hoc* test for multiple comparisons. A value of *p* < 0.05 was considered statistically significant. Multiplicity adjusted *p*-value was also assessed.

## 3. Results

### 3.1. Systematic review

**Figure 2:**
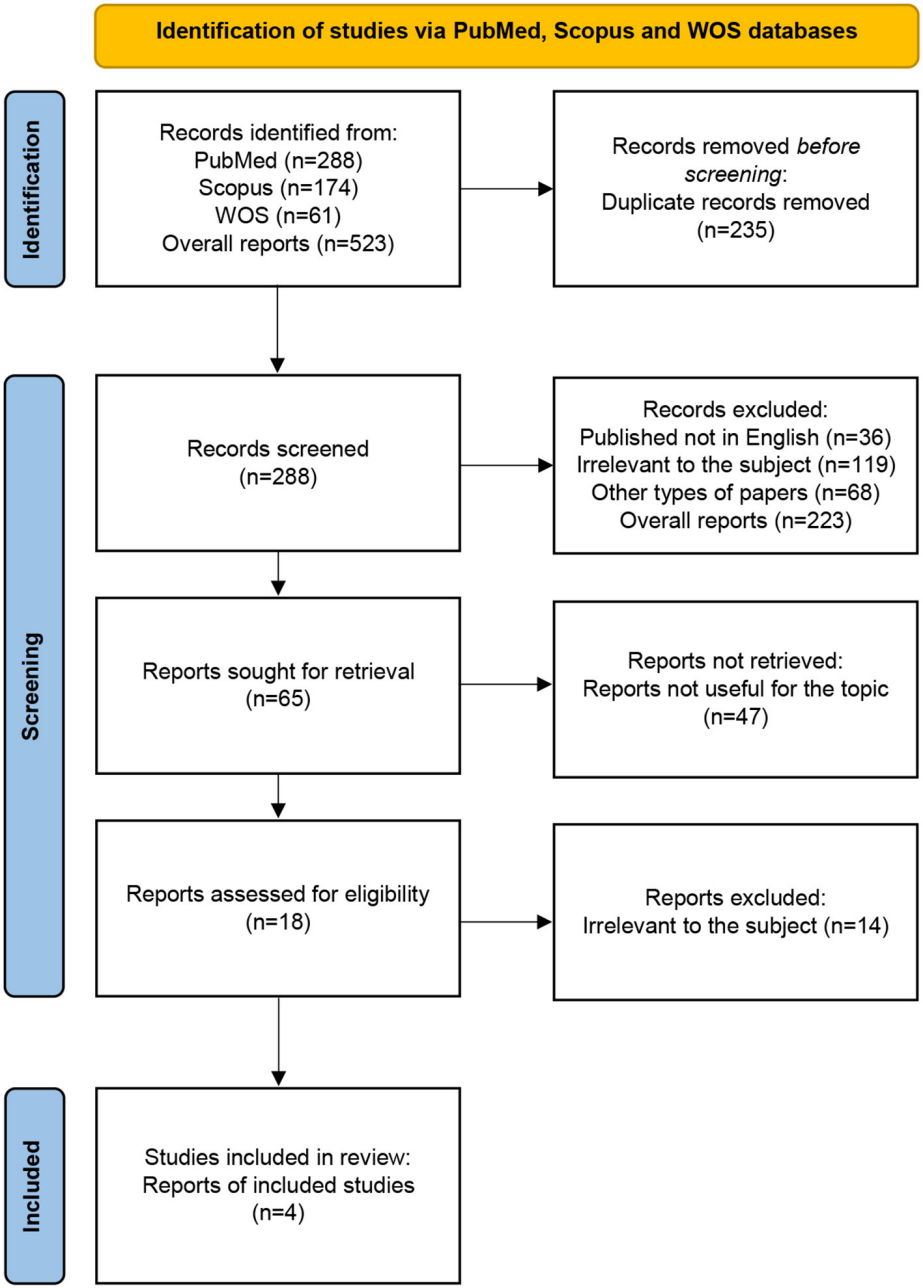
PRISMA flow diagram: systematic review methodology. WOS, Web of Science Core Collection.

The review of the bibliography led to the selection of 523 articles from the PubMed, Scopus, and WOS online databases; however, 235 of them were rejected due to the titles. A total of 228 abstracts were read; however, only 18 of them fit the inclusion and exclusion criteria. Other articles included 14 that were not related to the topic of our study. Eventually, four manuscripts were incorporated into the research (Figure 2). The results of our systemic review are presented in Table 2.

**Table 2:** Agomelatine's antiglycoxidative action in experimental and clinical research.

**Study design**	**Endpoints**	**References**
Streptozocin (STZ)-treated intraperitoneally male Sprague Dawley albino rats exposed to agomelatine (20 mg/kg/day)	Agomelatine inhibited STZ-induced oxidative damage of rat testicles by alleviating histological findings, decreasing malondialdehyde levels, as well as enhancing catalase and superoxide dismutase activities	(17)
Male Sprague Dawley rats injected intracerebroventricularly by STZ and after 3 months administrated agomelatine (40 mg/kg) for 30 days	Agomelatine restored STZ-enhanced β-amyloid level both in the frontal cortex, as well as in the hippocampus	(18)
Male Wistar rats intraperitoneally D-galactose-exposed treated with agomelatine (40 mg/kg/day)	Agomelatine downregulated the expression of the receptor for advanced glycation end products and also NADPH oxidase 2 and 4, inhibited ROS production, as well as stabilized mitochondrial membrane	(19)
A total of 40 depressed patients with cooccurring non-optimally controlled type 2 diabetes mellitus treated with sertraline (50–100 mg/day) or agomelatine (25–50 mg/day) for 4 months	Patients treated with agomelatine showed a reduction in anxiety and depression, as well as better self-care; there were no differences in fasting plasma glucose between the drugs, but agomelatine was more effective in reducing hemoglobin A1c levels; both study drugs were well tolerated by patients—none withdrew from the study	(20)

STZ, streptozocin.

### 3.2. Scavenging of reactive oxygen species

ROS are chemically active molecules that are formed in enzymatic or non-enzymatic oxidative reactions. Despite low concentrations, they are involved in many physiological processes. Increased ROS levels may lead to oxidative modifications of cellular biomolecules. Assessing the scavenging capacity of HO**•**, H_2_O_2_ and NO•–provides crucial information regarding the antioxidant properties of the test sample (35, 36).

#### 3.2.1. Scavenging of hydrogen peroxide

**Figure 3:**
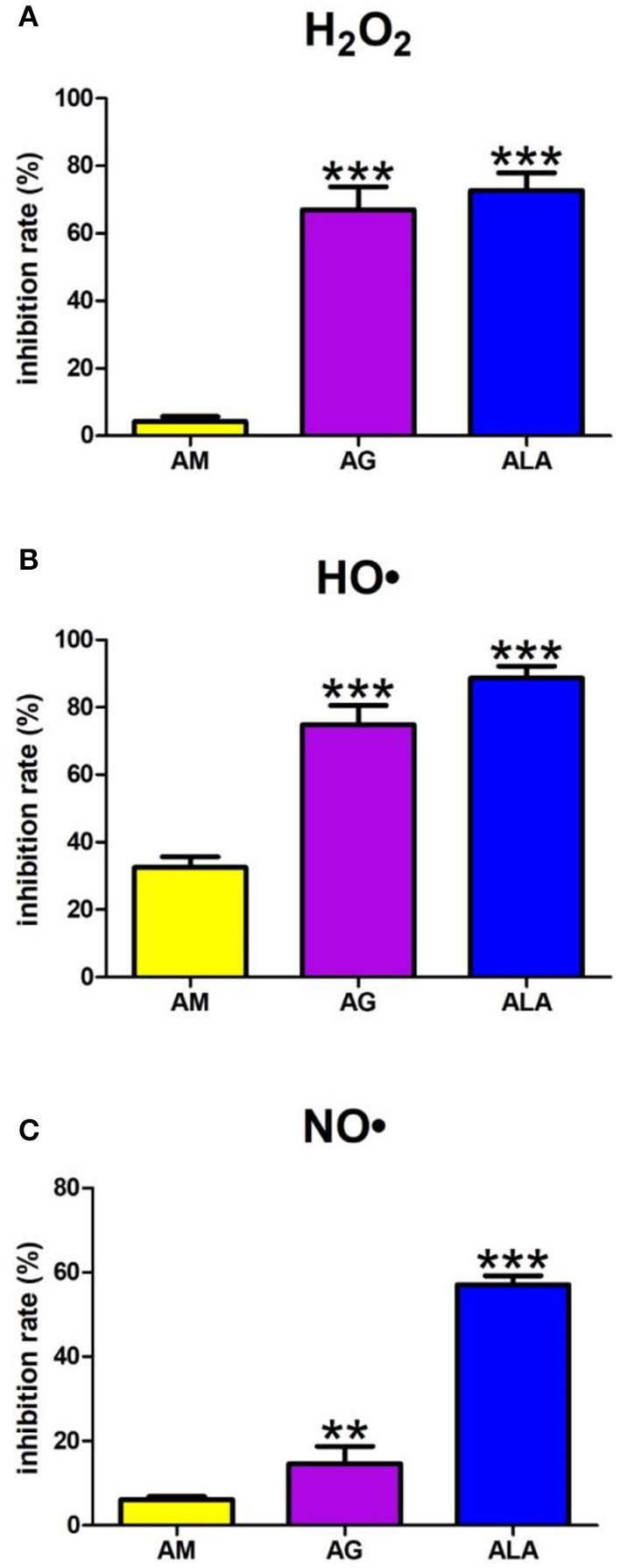
Scavenging of reactive oxygen species (ROS): hydrogen peroxide (H_2_O_2_) **(A)**, hydroxyl radical (HO•) **(B)** as well as nitric oxide (NO•) **(C)** by agomelatine and other additives. AG, aminoguanidine; ALA, α-lipoic acid; AM, agomelatine. **p < 0.01 vs. control (agomelatine); ***p < 0.001 vs. control (agomelatine).

A one-way ANOVA pointed out that the H_2_O_2_ scavenging inhibition rate differed between groups (*p* < 0.0001). Agomelatine scavenged H_2_O_2_ at 4%. The inhibition rate of H_2_O_2_ scavenging of aminoguanidine (+1,468%, *p* < 0.0001) and ALA (+1,600%, *p* < 0.0001) was significantly higher than the inhibition rate of agomelatine (Figure 3A).

#### 3.2.2. Scavenging of hydroxyl radical

The one-way ANOVA showed HO• scavenging capacity differed between the groups (*p* < 0.0001). The HO• scavenging inhibition rate of aminoguanidine (+130%, *p* < 0.0001) and ALA (+172%, *p* < 0.0001) was significantly higher than the inhibition rate of agomelatine (33%) (Figure 3B).

#### 3.2.3. Scavenging of nitric oxide

The one-way ANOVA presented that scavenging of NO• differed between groups (*p* < 0.0001). The NO• inhibition rate of agomelatine was 6%. Both aminoguanidine (+140%, *p* = 0.0014) and ALA (+835%, *p* < 0.0001) show significantly higher inhibition rates than agomelatine (Figure 3C).

### 3.3. Antioxidant activity

The antioxidant properties of a substance depend on the scavenging capacity of standard synthetic radicals (e.g., DPPH•) and their ability to chelate metals (e.g., FIC) (35, 37).

#### 3.3.1. Scavenging of 2,2-diphenyl-1-picrylhydrazyl radical

**Figure 4:**
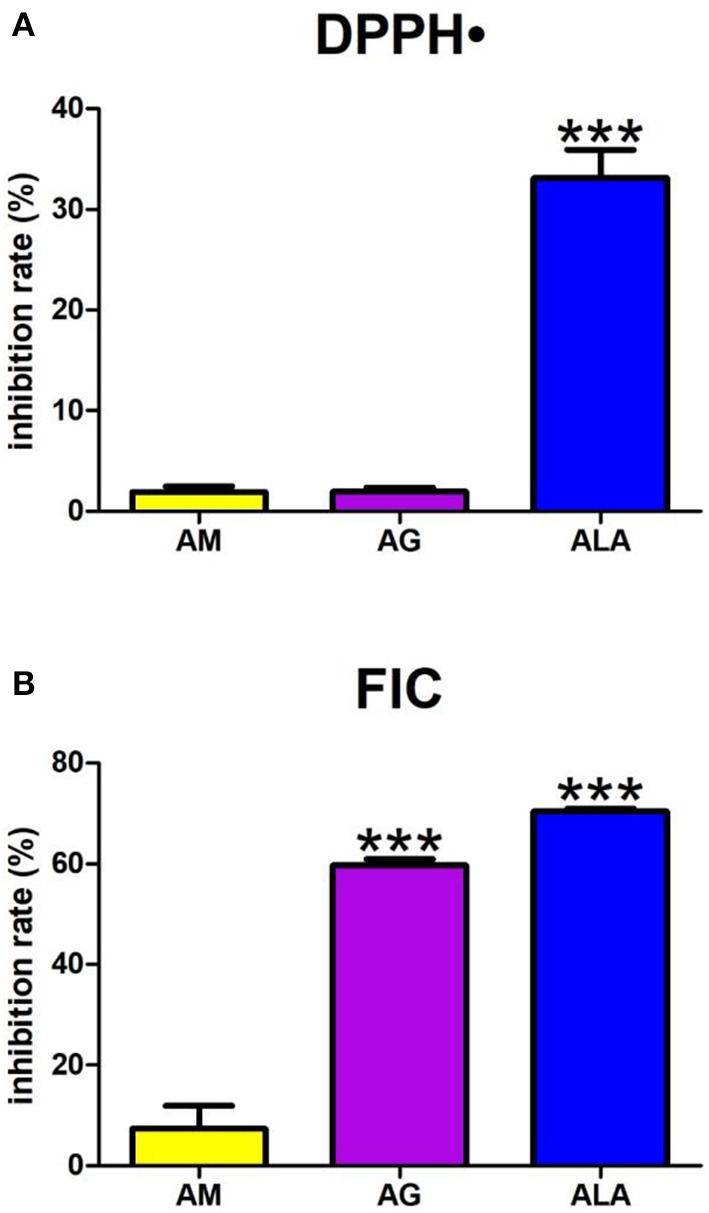
Antioxidant capacity: 2,2-diphenyl-1-picrylhydrazyl radical (DPPH•) reducing **(A)** as well as ferrous iron chelating (FIC) **(B)** by agomelatine and other additives. AG, aminoguanidine; ALA, α-lipoic acid; AM, agomelatine. ***p < 0.001 vs. control (agomelatine).

The one-way ANOVA revealed the scavenging of DPPH• differed between groups (*p* < 0.0001). The DPPH• scavenging capacity of ALA only was significantly elevated (+1,635%, *p* < 0.0001) compared to agomelatine (2%) (Figure 4A).

#### 3.3.2. Ferrous iron chelating

The one-way ANOVA demonstrated that FIC differed between groups (*p* < 0.0001). Agomelatine presented an FIC inhibition rate of 7%. This parameter was effectively elevated in aminoguanidine and ALA (+708%, *p* < 0.0001 and +853%, *p* < 0.0001, respectively) (Figure 4B).

### 3.4. Carbonyl and oxidative stress products in glucose-induced BSA glycation

#### 3.4.1. Carbonyl stress products

**Figure 5:**
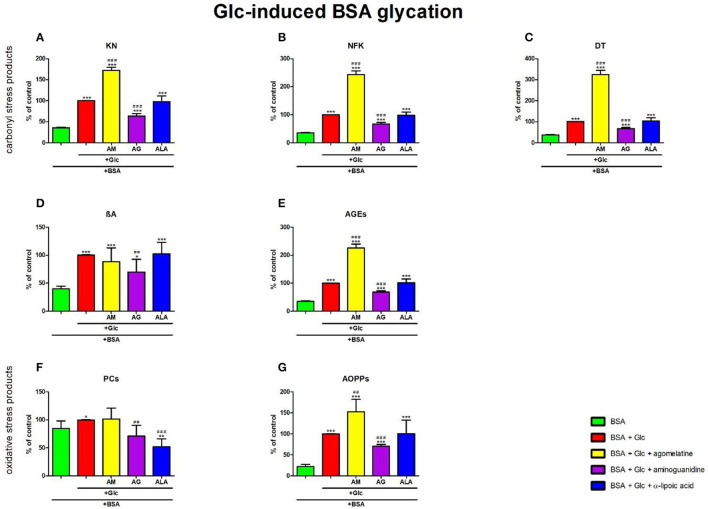
Effects of agomelatine addition to glucose (Glc)-glycated bovine serum albumin (BSA) on carbonyl stress products **(A–E)** and oxidative stress products **(F, G)**. AG, aminoguanidine; AGEs, advanced glycation end products; ALA, α-lipoic acid; AM, agomelatine; AOPPs, advanced oxidation protein products; βA, β-amyloid; BSA, bovine serum albumin; DT, dityrosine; Glc, glucose; KN, kynurenine; NFK, N-formylkynurenine; PCs, protein carbonyls. *p < 0.05 vs. negative control (BSA); **p < 0.01 vs. negative control (BSA); ***p < 0.001 vs. negative control (BSA); ^*##*^*p* < 0.01 vs. positive control (BSA + Glc); ^*###*^*p* < 0.001 vs. positive control (BSA + Glc).

The one-way ANOVA showed that KN contents differed between groups (*p* < 0.0001). The fluorescence of KN was elevated in Glc+agomelatine (+72%, *p* < 0.0001) but markedly lower in Glc+aminoguanidine (−36%, *p* < 0.0001), in comparison with Glc. The content of KN was significantly potentiated in Glc, Glc + agomelatine, Glc + aminoguanidine, and Glc + ALA (+183%, *p* < 0.0001; +387%, *p* < 0.0001; +80%, *p* < 0.0001; and +177%, *p* < 0.0001, respectively) compared to BSA (Figure 5A).

The one-way ANOVA found contents of NFK differed between groups (*p* < 0.0001). The fluorescence of NFK was higher in Glc+agomelatine (+143%, *p* < 0.0001) but lower in Glc+aminoguanidine (−33%, *p* < 0.0001) than in Glc alone. The content of NFK was well-augmented in Glc, Glc + agomelatine, Glc + aminoguanidine, and Glc + ALA (+183%, *p* < 0.0001; +586%, *p* < 0.0001; +90%, *p* < 0.0001; and +178%, *p* < 0.0001, respectively) vs. BSA (Figure 5B).

The one-way ANOVA revealed that the fluorescence of DT differed between the groups (*p* < 0.0001). The fluorescence of DT was relatively improved in Glc + agomelatine (+224%, *p* < 0.0001) compared to Glc alone. The biomarker was considerably decreased in Glc + aminoguanidine (−32%, *p* < 0.0001) vs. Glc. The content of DT was markedly elevated in Glc, Glc+agomelatine, Glc+aminoguanidine, and Glc+ALA (+165%, *p* < 0.0001; +757%, *p* < 0.0001; +79%, *p* < 0.0001; and +176%, *p* < 0.0001, respectively) when compared to BSA (Figure 5C).

The one-way ANOVA demonstrated βA contents differed between groups (*p* < 0.0001). The fluorescence of βA was decreased in Glc+aminoguanidine (−30%, *p* = 0.0095), compared to Glc alone. The content of βA was significantly augmented in Glc, Glc + agomelatine, Glc + aminoguanidine, and Glc + ALA (+150%, *p* < 0.0001; +121%, *p* = 0.0008; +74%, *p* = 0.0117; as well as +156%, *p* < 0.0001, respectively) vs. BSA (Figure 5D).

The one-way ANOVA indicated that the fluorescence of AGEs differed between groups (*p* < 0.0001). The AGEs fluorescence was elevated in Glc + agomelatine but markedly decreased in Glc + aminoguanidine (+126%, *p* < 0.0001 and −32%, *p* < 0.0001, respectively) when compared to Glc. The content of AGEs was well-augmented in Glc, Glc+agomelatine, Glc+aminoguanidine, and Glc + ALA (+192%, *p* < 0.0001; +559%, p < 0.0001; +98%, *p* < 0.0001; and +196%, p < 0.0001, respectively) vs. BSA (Figure 5E).

#### 3.4.2. Oxidative stress products

The one-way ANOVA reported that PC levels differed between groups (*p* < 0.0001). The concentration of PCs was also markedly decreased Glc+aminoguanidine and Glc+ALA (−29%, *p* = 0.0043 and −48%, *p* < 0.0001, respectively) vs. Glc. However, Glc (+18%, *p* = 0.0207) was considerably augmented, unlike Glc+ALA (-39%, *p* = 0.0038), compared to BSA (Figure 5F).

The one-way ANOVA pointed out that concentrations of AOPPs differed between groups (*p* < 0.0001). The level of AOPPs was elevated in Glc+agomelatine (+53%, *p* = 0.0015) but substantially lowered in Glc+aminoguanidine (−29%, *p* < 0.0001) vs. Glc. The concentration of AOPPs was markedly potentiated in Glc, Glc+agomelatine, Glc+aminoguanidine, and Glc+ALA (+346%, *p* < 0.0001; +580%, *p* < 0.0001; +215%, *p* < 0.0001; and +349%, *p* = 0.0004, respectively) compared to BSA (Figure 5G).

### 3.5. Carbonyl and oxidative stress products in fructose-induced BSA glycation

#### 3.5.1. Carbonyl stress products

**Figure 6:**
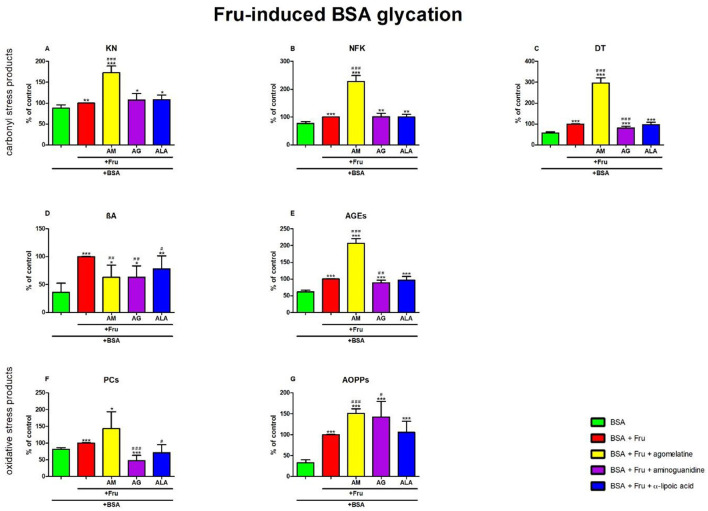
Effects of agomelatine addition to fructose (Fru)-glycated bovine serum albumin (BSA) on carbonyl stress products **(A–E)** and oxidative stress products **(F, G)**. AG, aminoguanidine; AGEs, advanced glycation end products; ALA, α-lipoic acid; AM, agomelatine; AOPPs, advanced oxidation protein products; βA, β-amyloid; BSA, bovine serum albumin; DT, dityrosine; Fru, fructose; KN, kynurenine; NFK, N-formylkynurenine; PCs, protein carbonyls. *p < 0.05 vs. negative control (BSA); **p < 0.01 vs. negative control (BSA); ***p < 0.001 vs. negative control (BSA); ^#^*p* < 0.05 vs. positive control (BSA + Fru); ^*##*^*p* < 0.01 vs. positive control (BSA + Fru); ^*###*^*p* < 0.001 vs. positive control (BSA + Fru).

The one-way ANOVA showed that KN contents differed between groups (*p* < 0.0001). The fluorescence of KN was only elevated in Fru +agomelatine (+73%, *p* < 0.0001) in comparison with Fru alone. The content of KN was significantly potentiated in Fru, Fru + agomelatine, Fru + aminoguanidine, and Fru + ALA (+14%, *p* = 0.0046; +96%, *p* < 0.0001; +22%, *p* = 0.0326; and +23%, *p* = 0.0105, respectively) than in BSA (Figure 6A).

The one-way ANOVA found contents of NFK differed between groups (*p* < 0.0001). The fluorescence of NFK was significantly elevated in Fru + agomelatine (+128%, *p* < 0.0001) in comparison with Fru alone. The content of NFK was substantially elevated in Fru (+29%, *p* < 0.0001), Fru + agomelatine (+193%, *p* < 0.0001), Fru + aminoguanidine (+30%, *p* = 0.0045), and Fru + ALA (+29%, *p* = 0.0015) compared to BSA (Figure 6B).

The one-way ANOVA revealed that the fluorescence of DT differed between groups (*p* < 0.0001). DT fluorescence was significantly elevated in Fru+agomelatine (+196%, *p* < 0.0001) but markedly decreased in Fru + aminoguanidine (−18%, *p* = 0.0002) in comparison with Fru. The content of DT was considerably elevated in Fru (+75%, *p* < 0.0001), Fru + agomelatine (+418%, *p* < 0.0001), Fru + aminoguanidine (+43%, *p* = 0.0003), and Fru + ALA (+70%, *p* < 0.0001) than in BSA (Figure 6C).

The one-way ANOVA demonstrated βA contents differed between groups (*p* < 0.0001). The fluorescence of βA was substantially decreased in Fru + agomelatine (−37%, *p* = 0.0024), Fru + aminoguanidine (−37%, *p* = 0.0015), and (−22%, *p* = 0.0431) vs. Fru alone. The content of βA was significantly augmented in Fru (+178%, *p* < 0.0001), Fru + agomelatine (+75%, *p* = 0.0444), Fru + aminoguanidine (+75%, *p* = 0.0369), and Fru + ALA (+117%, *p* = 0.0063) vs. BSA (Figure 6D).

The one-way ANOVA indicated that the fluorescence of AGEs differed between groups (*p* < 0.0001). AGEs fluorescence was significantly elevated in Fru + agomelatine (+107%, *p* < 0.0001) but relevantly diminished in Fru + aminoguanidine (−12%, *p* = 0.0064) vs. Fru. The content of AGEs was higher in Fru (+63%, *p* < 0.0001), Fru + agomelatine (+237%, *p* < 0.0001), Fru + aminoguanidine (+44%, *p* = 0.0001), and Fru + ALA (+58%, *p* < 0.0001) than in BSA (Figure 6E).

#### 3.5.2. Oxidative stress products

The one-way ANOVA reported that the PC levels differed between groups (*p* < 0.0001). The concentration of PCs was considerably decreased in Fru + aminoguanidine (−53%, *p* < 0.0001) and Fru + ALA (−29%, *p* = 0.0151) in comparison with Fru alone. The level of PCs was significantly augmented in Fru (+23%, *p* < 0.0001) and Fru+agomelatine (+75%, *p* = 0.0147) vs. BSA. The parameter was lower in Fru+aminoguanidine (−42%, *p* = 0.0005) than in BSA (Figure 6F).

The one-way ANOVA pointed out that concentrations of AOPPs differed between groups (*p* < 0.0001). The concentration of AOPPs was markedly elevated in Fru + agomelatine (+51%, *p* < 0.0001) and Fru + aminoguanidine (+42%, *p* = 0.0196) vs. Fru. The level of AOPPs was significantly augmented in Fru (+200%, *p* < 0.0001), Fru + agomelatine (+353%, *p* < 0.0001), Fru + aminoguanidine (+327%, *p* = 0.0002), and Fru+ALA (+216%, *p* = 0.0002) in comparison with BSA (Figure 6G).

### 3.6. Carbonyl and oxidative stress products in galactose-induced BSA glycation

#### 3.6.1. Carbonyl stress products

**Figure 7:**
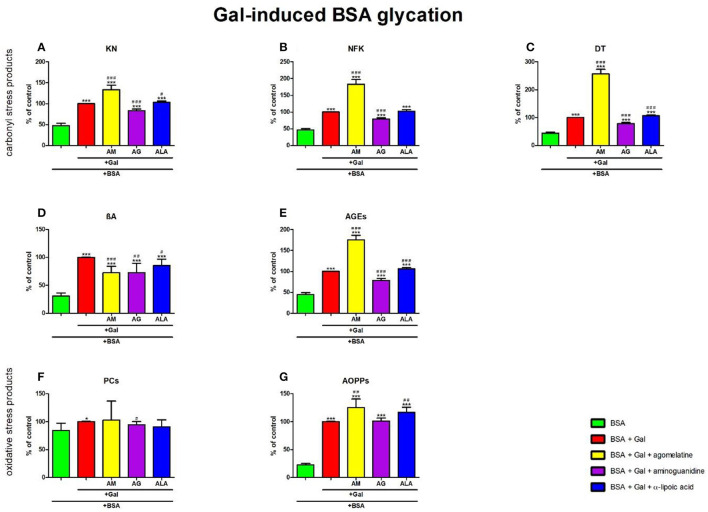
Effects of agomelatine addition to galactose (Gal)-glycated bovine serum albumin (BSA) on carbonyl stress products **(A–E)** and oxidative stress products **(F, G)**. AG, aminoguanidine; AGEs, advanced glycation end products; ALA, α-lipoic acid; AM, agomelatine; AOPPs, advanced oxidation protein products; βA, β-amyloid; BSA, bovine serum albumin; DT, dityrosine; Gal, galactose; KN, kynurenine; NFK, N-formylkynurenine; PCs, protein carbonyls. *p < 0.05 vs. negative control (BSA); **p < 0.01 vs. negative control (BSA); ***p < 0.001 vs. negative control (BSA); ^#^*p* < 0.05 vs. positive control (BSA + Gal); ^*##*^*p* < 0.01 vs. positive control (BSA + Gal); ^*###*^*p* < 0.001 vs. positive control (BSA + Gal).

The one-way ANOVA showed that the KN contents differed between groups (*p* < 0.0001). The fluorescence of KN was relatively elevated in Gal + agomelatine (+34%, *p* < 0.0001) and Gal + ALA (+3%, *p* = 0.0246) vs. Gal alone. This parameter was considerably decreased in Gal + aminoguanidine (−87%, *p* < 0.0001) vs. Gal. The content of KN was substantially attenuated in Gal (+109%, *p* < 0.0001), Gal + agomelatine (+180%, *p* < 0.0001), Gal + aminoguanidine (+74%, *p* < 0.0001), and Gal + ALA (+116%, *p* < 0.0001) vs. BSA (Figure 7A).

The one-way ANOVA found the contents of NFK differed between groups (*p* < 0.0001). The fluorescence of NFK was relevantly improved in Gal+agomelatine (+83%, *p* < 0.0001) compared to Gal alone. The biomarker was considerably decreased in Gal + aminoguanidine (−20%, *p* < 0.0001) vs. Gal. The content of NFK was considerably high in Gal, Gal + agomelatine, Gal + aminoguanidine, and Gal + ALA in comparison with Gal alone (+116%, *p* < 0.0001; +296%, *p* < 0.0001; +72%, *p* < 0.0001; and +122%, *p* < 0.0001, respectively) in comparison with BSA (Figure 7B).

The one-way ANOVA revealed that the fluorescence of DT differed between groups (*p* < 0.0001). The fluorescence of DT was markedly increased both in Gal + agomelatine and Gal + ALA (+157%, *p* < 0.0001 and +7%, *p* = 0.0002, respectively) vs. Gal alone. This parameter was relevantly decreased in Gal + aminoguanidine (−21%, *p* < 0.0001) vs. Gal. The content of DT was significantly augmented in Gal (+128%, *p* < 0.0001), Gal + agomelatine (+486%, *p* < 0.0001), Gal + aminoguanidine (+80%, *p* < 0.0001), and Gal + ALA (+144%, *p* < 0.0001) vs. BSA (Figure 7C).

The one-way ANOVA demonstrated βA contents differed between groups (*p* < 0.0001). The fluorescence of βA was markedly decreased in Gal + agomelatine, Gal + aminoguanidine, and Gal + ALA vs. Gal (−27%, *p* = 0.0002; −27%, *p* = 0.0028; and −15%, *p* = 0.0107, respectively). The content of βA was significantly augmented in Gal (+224%, *p* < 0.0001), Gal + agomelatine (+135%, *p* < 0.0001), Gal + aminoguanidine (+136%, *p* = 0.0002), and Gal + ALA (+176%, *p* < 0.0001) vs. BSA (Figure 7D).

The one-way ANOVA indicated that the fluorescence of AGEs differed between groups (*p* < 0.0001). The fluorescence of AGEs was markedly elevated in Gal + agomelatine (+75%, *p* < 0.0001) and Gal + ALA (+6%, *p* = 0.0009) compared to Gal. The marker was relevantly decreased in Gal + aminoguanidine (−22%, *p* < 0.0001) vs. Gal alone. The content of AGEs was considerably elevated in Gal, Gal+agomelatine, Gal + aminoguanidine, and Gal + ALA vs. BSA (+124%, *p* < 0.0001, +293%, *p* < 0.0001, +76%, *p* < 0.0001, and +139%, *p* < 0.0001, respectively) (Figure 7E).

#### 3.6.2. Oxidative stress products

The one-way ANOVA reported that the PC levels did not differ between the groups (*p* = 0.3964).

The one-way ANOVA pointed out that the concentrations of AOPPs differed between the groups (*p* < 0.0001). The concentration of AOPPs was elevated in Gal + agomelatine and Gal + ALA (+25%, *p* = 0.0024 and +17%, *p* = 0.0011, respectively) vs. Gal alone. The level of AOPPs was significantly potentiated in Gal (+343%, *p* < 0.0001), Gal + agomelatine (+455%, *p* < 0.0001), Gal + aminoguanidine (+347%, *p* < 0.0001), and Gal + ALA (+418%, *p* < 0.0001) compared to BSA (Figure 7G).

### 3.7. Carbonyl and oxidative stress products in glyoxal-induced BSA glycation

#### 3.7.1. Carbonyl stress products

**Figure 8:**
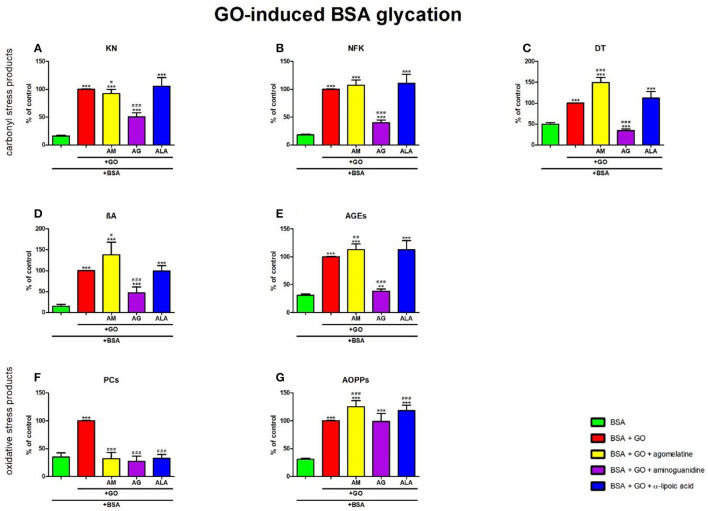
Effects of agomelatine addition to glyoxal (GO)-glycated bovine serum albumin (BSA) on carbonyl stress products **(A–E)** and oxidative stress products **(F, G)**. AG, aminoguanidine; AGEs, advanced glycation end products; ALA, α-lipoic acid; AM, agomelatine; AOPPs, advanced oxidation protein products; βA, β-amyloid; BSA, bovine serum albumin; DT, dityrosine; GO, glyoxal; KN, kynurenine; NFK, N-formylkynurenine; PCs, protein carbonyls. **p < 0.01 vs. negative control (BSA); ***p < 0.001 vs. negative control (BSA); ^#^*p* < 0.05 vs. positive control (BSA + GO); ^*##*^*p* < 0.01 vs. positive control (BSA + GO); ^*###*^*p* < 0.001 vs. positive control (BSA + GO).

The one-way ANOVA showed that the KN contents differed between groups (*p* < 0.0001). KN fluorescence in GO+agomelatine (−8%, *p* = 0.0298) and GO+aminoguanidine (−49%, *p* < 0.0001) was relevantly diminished vs. GO. The content of KN was considerably elevated in GO (+518%, *p* < 0.0001), GO + agomelatine (+469%, *p* < 0.0001), GO + aminoguanidine (+213%, *p* < 0.0001), and GO + ALA (+553%, *p* < 0.0001) over against BSA (Figure 8A).

The one-way ANOVA found the contents of NFK differed between groups (*p* < 0.0001). NFK fluorescence in GO + aminoguanidine was substantially diminished vs. GO alone (−60%, *p* < 0.0001). The content of NFK was substantially potentiated in GO (+454%, *p* < 0.0001), GO + agomelatine (+495%, *p* < 0.0001), GO + aminoguanidine (+121%, *p* < 0.0001), and GO + ALA (+513%, *p* < 0.0001) compared to BSA (Figure 8B).

The one-way ANOVA revealed that the fluorescence of DT differed between groups (*p* < 0.0001). The fluorescence of DT was relevantly improved in GO + agomelatine (+50%, *p* < 0.0001) compared to GO alone. The biomarker was considerably decreased in GO + aminoguanidine (−65%, *p* < 0.0001) vs. GO. The content of DT was markedly potentiated in GO, GO + agomelatine, and GO + ALA (+100%, *p* < 0.0001; +200%, *p* < 0.0001; and +125%, *p* < 0.0001, respectively) vs. BSA. The biomarker was considerably decreased in GO + aminoguanidine (−31%, *p* < 0.0001) vs. BSA (Figure 8C).

The one-way ANOVA demonstrated βA contents differed between groups (*p* < 0.0001). The fluorescence of βA was markedly higher in GO + agomelatine (+38%, *p* = 0.0118) than in GO. This parameter was significantly decreased in GO+aminoguanidine (−53%, *p* < 0.0001) vs. GO alone. The content of βA was significantly potentiated in GO (+578%, *p* < 0.0001), GO + agomelatine (+834%, *p* < 0.0001), GO + aminoguanidine (+216%, *p* = 0.0004), and GO + ALA (+573%, *p* < 0.0001) vs. BSA (Figure 8D).

The one-way ANOVA indicated that the fluorescence of AGEs differed between groups (*p* < 0.0001). The fluorescence of AGEs was relevantly improved in GO + agomelatine (+13%, *p* = 0.0082) vs. GO. The biomarker was substantially lower in GO + aminoguanidine (−62%, *p* < 0.0001) vs. GO alone. The biomarker was effectively augmented in GO, GO + agomelatine, GO + aminoguanidine, and GO + ALA vs. BSA (+223%, *p* < 0.0001; +265%, *p* < 0.0001; +23%, *p* = 0.005; and +263%, *p* < 0.0001, respectively) (Figure 8E).

#### 3.7.2. Oxidative stress products

The one-way ANOVA reported that PC levels differed between groups (*p* < 0.0001). The concentration of PCs was markedly lower in GO + agomelatine, GO + aminoguanidine, and GO + ALA compared to GO (−68%, *p* < 0.0001; −73%, *p* < 0.0001; and −68%, *p* < 0.0001, respectively). The level of PCs was markedly boosted in GO (+186%, *p* < 0.0001) when compared to BSA (Figure 8F).

The one-way ANOVA pointed out that the concentrations of AOPPs differed between groups (*p* < 0.0001). The concentration of AOPPs was considerably elevated in GO + agomelatine (+26%, *p* = 0.0002) and GO + ALA (+18%, *p* = 0.0009) vs. GO alone. The level of AOPPs was significantly potentiated in GO, GO + agomelatine, GO + aminoguanidine, and GO + ALA vs. BSA (+225%, *p* < 0.0001; +307%, *p* < 0.0001; +221%, *p* < 0.0001; and +284%, *p* < 0.0001, respectively) (Figure 8G).

### 3.8. Carbonyl and oxidative stress products in methylglyoxal-induced BSA glycation

#### 3.8.1. Carbonyl stress products

**Figure 9:**
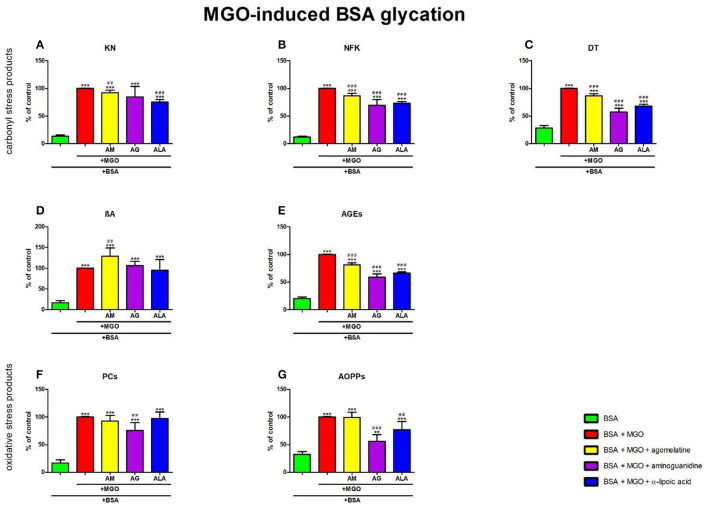
Effects of agomelatine addition to methylglyoxal (MGO)-glycated bovine serum albumin (BSA) on carbonyl stress products **(A–E)** and oxidative stress products **(F, G)**. AG, aminoguanidine; AGEs, advanced glycation end products; ALA, α-lipoic acid; AM, agomelatine; AOPPs, advanced oxidation protein products; βA, β-amyloid; BSA, bovine serum albumin; DT, dityrosine; KN, kynurenine; MGO, methylglyoxal; NFK, N-formylkynurenine; PCs, protein carbonyls. **p < 0.01 vs. negative control (BSA); ***p < 0.001 vs. negative control (BSA); ^*##*^*p* < 0.01 vs. positive control (BSA + MGO); ^*###*^*p* < 0.001 vs. positive control (BSA + MGO).

The one-way ANOVA showed that KN contents differed between groups (*p* < 0.0001). KN fluorescence in MGO + agomelatine and MGO + ALA was substantially diminished vs. MGO (−8%, *p* = 0.0018 and −25%, *p* < 0.0001, respectively). The content of KN was significantly potentiated in MGO (+631%, *p* < 0.0001), MGO + agomelatine (+573%, *p* < 0.0001), MGO + aminoguanidine (+519%, *p* < 0.0001), and MGO + ALA (+451%, *p* < 0.0001) compared to BSA (Figure 9A).

The one-way ANOVA found contents of NFK differed between groups (*p* < 0.0001). The fluorescence of NFK was markedly lower in MGO + agomelatine (−13%, *p* < 0.0001), MGO + aminoguanidine (−31%, *p* < 0.0001), and MGO + ALA (−27%, *p* < 0.0001), in comparison with MGO alone. The content of NFK was significantly potentiated in MGO (+726%, *p* < 0.0001), MGO + agomelatine (+617%, *p* < 0.0001), MGO + aminoguanidine (+474%, *p* < 0.0001), and MGO + ALA (+504%, *p* < 0.0001) compared to BSA (Figure 9B).

The one-way ANOVA revealed that the fluorescence of DT differed between groups (*p* < 0.0001). The fluorescence of DT was relevantly diminished in MGO + agomelatine, MGO + aminoguanidine, and MGO + ALA vs. MGO (−13%, *p* < 0.0001; −43%, *p* < 0.0001; and −32%, *p* < 0.0001, respectively). The content of DT was considerably attenuated in MGO (+250%, *p* < 0.0001), MGO + agomelatine (+204%, *p* < 0.0001), MGO + aminoguanidine (+101%, *p* < 0.0001), and MGO + ALA (+139%, *p* < 0.0001) when compared to BSA (Figure 9C).

The one-way ANOVA demonstrated βA contents differed between groups (*p* < 0.0001). The fluorescence of βA was relatively elevated in MGO + agomelatine (+29%, *p* = 0.0051) in comparison with MGO alone. The content of βA was markedly enhanced in MGO, MGO + agomelatine, MGO + aminoguanidine, and MGO+ALA (+506%, *p* < 0.0001; +680%, *p* < 0.0001; +544%, *p* < 0.0001; and +476%, *p* < 0.0001, respectively) in comparison with BSA (Figure 9D).

The one-way ANOVA indicated that the fluorescence of AGEs differed between groups (*p* < 0.0001). The fluorescence of AGEs in MGO + agomelatine (−18%, *p* < 0.0001), MGO + aminoguanidine (−41%, *p* < 0.0001), and MGO + ALA (−34%, *p* < 0.0001) was relevantly diminished vs. MGO. However, the content of AGEs was markedly elevated in MGO, MGO + agomelatine, MGO + aminoguanidine, and MGO + ALA vs. BSA (+393%, *p* < 0.0001; +302%, *p* < 0.0001; +189%, *p* < 0.0001; and +226%, *p* < 0.0001, respectively) (Figure 9E).

#### 3.8.2. Oxidative stress products

The one-way ANOVA reported that PC levels differed between groups (*p* < 0.0001). The concentration of PCs was markedly lowered in MGO + aminoguanidine (−24%, *p* = 0.002) vs. MGO alone. The level of PCs was relevantly higher in MGO (+485%, *p* < 0.0001), MGO + agomelatine (+445%, *p* < 0.0001), MGO + aminoguanidine (+343%, *p* < 0.0001), and MGO + ALA (+471%, *p* < 0.0001) when compared to BSA (Figure 9F).

The one-way ANOVA pointed out that the concentrations of AOPPs differed between groups (*p* < 0.0001). The concentration of AOPPs was relevantly diminished in MGO + aminoguanidine and MGO + ALA vs. MGO (−44%, *p* < 0.0001 and −23%, *p* = 0.0041, respectively). The biomarker was effectively augmented in MGO, MGO + agomelatine, MGO + aminoguanidine, and MGO + ALA in comparison with BSA (+210%, *p* < 0.0001; +208%, *p* < 0.0001; +73%, *p* = 0.0014; and +139%, *p* < 0.0001, respectively) (Figure 9G).

### 3.9. Binding affinity analysis

**Figure 10:**
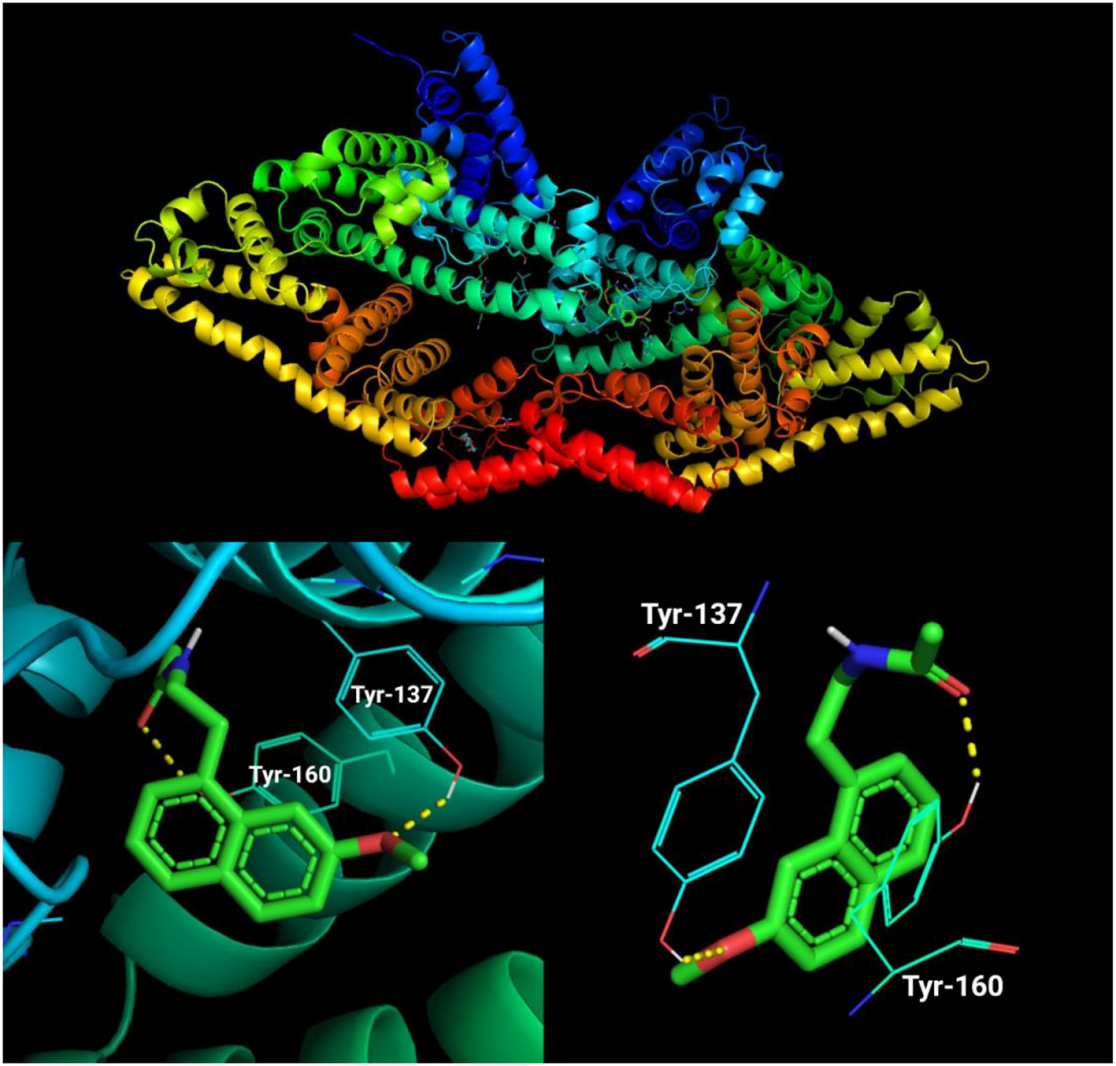
Visualization of an agomelatine docking site (mode 1) in a BSA particle.

The molecular docking simulation of agomelatine indicated its low affinity to a BSA particle with a score of −8 kcal/mol. The sole docking site had root-mean-square deviations of atomic positions (RMSD) below 3, which revealed polar contact with tyrosines at positions 137 and 160 of the BSA particle chain (Table 3; Figure 10) (38, 39).

**Table 3:** Molecular docking simulation of agomelatine to BSA results.

**Mode**	**Affinity (kcal/mol)**	**RMSD (lower bond)**	**RMSD (upper bond)**	**Amino acid residues**
1	−8.0	0.000	0.000	Tyr-137, Tyr-160
2	−8.0	2.366	4.355	Arg-185
3	−7.5	24.450	26.418	Arg-185
4	−7.4	4.821	6.194	Arg-185
5	−7.4	2.672	4.920	Tyr-160
6	−7.1	2.849	4.113	Lys-116
7	−7.1	23.682	25.774	Tyr-160
8	−7.0	2.951	7.416	Lys-136
9	−6.9	23.207	25.530	Asp-118

Arg, arginine; Asp, aspartic acid; Lys, lysine; RMSD, root-mean-square deviations of atomic positions; Tyr, tyrosine.

## 4. Discussion

The antidepressant mechanism of agomelatine action is not yet completely understood (6, 7, 40–42). Since molecular studies of the antiglycation/antioxidant properties of agomelatine have yet to be conducted, our study was the first to evaluate them comprehensively. We showed that agomelatine does not protect against protein glycation and oxidation in *in vitro* models. The drug also showed no antioxidant capacity.

Protein glycation is the process of non-enzymatic reaction of reducing substances (sugars, aldehydes, and/or ketones) with free amino groups of proteins (43). Protein glycation proceeds in multiple steps (collectively called a Maillard reaction) and begins with the reaction of carbonyl groups of glycation agents with proteins rich in free amino groups. The most susceptible to glycation are proteins with high amounts of lysine (Lys), cysteine (Cys), and arginine (Arg) (44). As a result of Amadori rearrangement, early products of protein glycation (APs, KN, NFK, and DT) are formed (45). Degradation of APs and the subsequent oxidation, dehydration, polymerization, and condensation leads to the formation of late protein glycation products (including βA and AGEs) (46, 47). During these reactions, ROS are also produced that modify amino acids, transform prosthetic groups of proteins, and induce their fragmentation or aggregation (48). Modifications of Lys increase its affinity for Cu^2+^ and Fe^2+^ ions as well as other oxidants in Fenton and Haber–Weiss reactions. In addition, sugars in their high concentrations undergo auto-oxidation, which also causes ROS formation (49, 50). Interestingly, both intermediate (PCs) and final oxidation protein products (AOPPs) intensify the process of protein glycation (51). Since glycation and oxidation potentiate each other, they are sometimes referred to as “glycoxidation” (52). Albumin is especially susceptible to glycoxidation due to its high plasma concentration, relatively long (~20 days) half-life, as well as high content of Lys, Cys, and Arg residues (49, 53). In addition to the structural similarity compared with its human counterpart (human serum albumin) and associated analogous binding properties, BSA has good stability, low cost, and wide availability. For this reason, the BSA model is often used in laboratory experiments (49, 53–55).

Sugars and aldehydes differ in the mechanism and kinetics of BSA glycation. While MGO glycates, predominantly Arg and His, are contained in the BSA structure, Glc shows affinity mainly for Lys at positions 534 and 232 (30% of the total BSA glycation) (56–59). Thus, to objectively evaluate the antiglycation properties of agomelatine, we decided to use various glycation factors such as Glc, Fru, Gal, GO, and MGO (21, 22, 26). In our experiment, all the agents used induced glycation (↑KN, ↑NFK, ↑DT, ↑βA, and ↑AGEs) and oxidation (↑PCs and ↑AOPPs) during their coincubation with BSA. Carbonyl/oxidative stress-caused modifications disrupt the biological function of albumin. They contribute to the reduced affinity for transported drugs and biological compounds (proximity of binding sites), reduced antioxidant properties (modification of Cys-34 and N-terminus), increased prooxidant activity (cross-reactions of oxidized albumin with other proteins, increased affinity of altered Lys for transition metal ions), and shortened protein half-life to 14 days, increasing its susceptibility to proteolysis (49, 53, 60–63).

The formed AGEs and AOPPs aggregate and accumulate in the central nervous system (64, 65). The energy metabolism of nerve cells is based on glucose, by which neurons easily undergo glycation- and ROS-mediated damage (11). Chronic emotional stress contributes to the increased production of ROS and decreased levels of antioxidants (66). Neuronal redox imbalance results in decreased ATP concentration, the inhibition of glycolysis, and the promotion of AGEs formation (67). AGEs/AOPPs combined with RAGE cause the activation of NOX and nuclear factor kappa-light-chain-enhancer of activated B cells (NF-κB). The consequence is the increased transcription of proinflammatory factors (including cytokines, chemokines, adhesion molecules, growth factors, cyclooxygenase 2, and NO• synthases) and the overproduction of ROS (68–70). Many metabolites of the tryptophan-kynurenine pathway also modulate neuroplasticity and exert neurotoxic activity (71). In part, this effect is exerted through the impact on NMDA signaling or glutamatergic neurotransmission (71, 72). The tyrosine metabolite, DT, is involved in the cross-linking of βA (73). It was shown that cerebral accumulation of βA damages neurons and promotes synaptic membrane depolarization, mitochondrial impairment, and high calcium influx (74). Finally, chronic inflammation, mitochondrial dysfunction, and oxidative stress lead to apoptotic and necrotic neuronal death, resulting in clinical symptoms of depression (74–76). A similar mechanism of neuronal damage is postulated in the course of neurodegenerative diseases (77, 78). Some of them (e.g., Alzheimer's disease, Huntington's chorea, or Parkinson's disease) contribute to the development of dementia. Therefore, glycoxidation plays a key role in the etiopathogenesis of both depression and dementia (66, 77–81).

Agomelatine did not prevent carbonyl stress, and in some cases, the drug even intensified protein glycation. Agomelatine caused an increase in KN, NFK, DT, and AGEs fluorescence of BSA glycated by Glc, Fru, and Gal. The drug also increased DT and AGEs in GO-induced BSA glycation. Moreover, the content of βA in BSA incubated with both GO and MGO increased under the influence of agomelatine. Thus, the studied drug showed a proglycation effect. In contrast, the model substances, aminoguanidine (a glycation inhibitor) and ALA (an antioxidant), prevented these processes significantly. Aminoguanidine counteracts carbonyl stress by neutralizing α,β-dicarbonyl compounds. In addition, the drug has an antioxidant effect due to the guanidinium group—it reacts with HO• and superoxide radicals (82). ALA and dihydro-lipoic acid (DHLA; the reduced form of ALA) are ROS scavengers. They also cause restoration of low molecular weight antioxidants such as reduced glutathione and vitamin E (44).

In molecular docking analysis, agomelatine showed a very weak (−8 kcal/mol) affinity to the BSA molecule. Peyrin et al. (83) postulate a two-step interaction between a ligand and albumin. In the first step, the ligand approaches the hydrophobic cavity of the albumin. Drugs can bind to non-specific sites on the hydrophobic albumin surface with an affinity depending on their hydrophobicity (regardless of their chemical structure). Specific attachment is characterized by very high affinity and low binding capacity (83). On the other hand, non-specific interaction shows low affinity with unlimited ligand binding capacity (83–85). Pharmacokinetic studies of agomelatine have shown that this drug binds more than 95% to plasma proteins (8, 40). A low affinity and high attachment capacity are features of non-specific binding (84, 85). Ligand attachment can change the tertiary structure of the albumin (83). In this way, the drug can enhance the attachment of glycation molecules to albumin, exacerbating carbonyl stress.

Antiglycation properties of agomelatine can be dependent on the antioxidant capacity. Hence we also evaluated its ability to iron chelation, reduction of synthetic DPPH• and scavenging of ROS which are physiologically generated in biological systems. Agomelatine failed to show antioxidant activity in the FIC and DPPH• tests. Agomelatine did not scavenge H_2_O_2_ or NO•. It only weakly scavenged HO• (more than two times as weak as aminoguanidine and almost three times as weak as ALA). Therefore, the drug did not show antioxidant activity.

However, all articles retrieved from the systematic review (three experimental and one clinical *in vivo* studies) reported the prevention of carbonyl/oxidative stress. Agomelatine counteracted lipid peroxidation, ROS generation, βA, and hemoglobin A1c formation, decreased the expression of RAGE as well as NOX-2 and −4. On the contrary, it increased the activity of enzymatic antioxidants (catalase and superoxide dismutase) and also stabilized the mitochondrial potential (via restoration of Bax/Bcl2 balance) (17–20). Despite the lack of antiglycoxidative properties *per se*, agomelatine may alleviate carbonyl/oxidative stress by improving brain metabolism. The drug can stimulate neuronal protective mechanisms (such as increasing the activity of enzymatic antioxidants). Additionally, Ilieva et al. (86) reported the agomelatine-mediated reduction of frontal cortical and hippocampal levels of proinflammatory cytokines (TNF-α and IL-1β) in the Alzheimer's disease rat model. The authors also report a protective effect of chronic agomelatine treatment on neurons in the temporal CA3b field of the hippocampus and the temporal piriform cortex (86). Demir Özkay et al. (87) showed that the drug increased the number and volume of pyramidal neurons as well as granular neurons in the dentate gyrus and CA1-3 hippocampal subregions in old rats. Agomelatine also caused an increase in the number of more stable types of dendritic spines (mushroom and stubby), which may indicate an improvement in adaptive capacity (87). Molteni et al. (88) found that agomelatine attenuated the LPS-induced increase in levels of the proinflammatory cytokines IL-1β and interleukin-6 (IL-6) in the rat brain by inhibiting NF-κB translocation and modulating microglia activation. In addition, the drug modulated the expression of tryptophan-kynurenine pathway enzymes thought to be important in the pathogenesis of depression associated with inflammation. In this way, agomelatine may nullify the negative effects on the brain of the glycoxidation process involved in the onset of depression (88). Such an effect at the tissue/organ level can be found in animal models or clinical trials (as confirmed by the conducted systematic review). Agomelatine's metabolites could also potentially account for the actions of its antioxidant and antiglycation action *in vivo*. Agomelatine is metabolized in the liver, 90% by the isoenzyme CYP1A2, and 10% by CYP2C9 and CYP2C19 (89, 90). None of the four main metabolites of agomelatine (7-desmethyl agomelatine, 3-hydroxyagomelatine, dihydrodiol-agomelatine, and desacetamide-agomelatine-carboxylic acid) exerts antidepressant effects; however, at least one may prevent protein glycation/oxidation. This is most likely to be the case with 7-desmethyl agomelatine, which passes through the blood–brain barrier to a high degree, as does the parent compound (91, 92). Therefore, further studies on evaluating the antiglycoxidative properties of agomelatine metabolites are needed.

The number of previous studies related to agomelatine's antiglycoxidative properties is limited, and they assessed only single parameters of carbonyl or oxidative stress. In this study, we are the first to evaluate exhaustively the antiglycation and antioxidant potential of agomelatine. The drug did not show antioxidant properties, while it had a proglycation effect in *in vitro* assays. A literature review suggests that agomelatine may improve brain metabolism (e.g., by stimulating defense mechanisms, thereby enhancing antiglycoxidative potential) or prevent carbonyl/oxidative stress through its metabolites. Therefore, it becomes necessary to conduct further studies to determine the as-yet-unknown mechanisms of agomelatine's therapeutic effects.

## Data availability statement

Publicly available datasets were analyzed in this study. This data can be found here: https://pubchem.ncbi.nlm.nih.gov/compound/Agomelatine.

## Author contributions

MN performed laboratory determinations, performed the statistical analysis, interpreted the data, prepared the graphic part of the manuscript, and wrote the manuscript. KL performed laboratory determinations and wrote the manuscript. MŻ-P and JŁ interpreted the data. AZ conceptualized and reviewed the article and gave final approval for the version to be published. MM conceptualized the article, performed laboratory determinations, interpreted the data, prepared the graphic part of the manuscript, wrote the manuscript, reviewed the article, and granted final approval of the version to be published. All authors contributed to the article and approved the submitted version.
